# Progress in Genito-Urinary Surgery

**Published:** 1902-05-24

**Authors:** 


### Progress in Genito-Urinary Surgery.

(*Continued from page* 117.)

**The Treatment of Syphilis.**—Winfield Ayres[12] has written on this subject. He points out that treatment with mercury was begun over 400 years ago. The disease is so insidious and takes so long to eradicate that whatever preparation is selected to deal with it must be one which is easily borne and will not cause any general disturbance. Those who believe that the constant, as distinguished from the interrupted treatment is the best, systematically prescribe mercury for three years. Ayres is certain that the full dose of mercury should be constantly given for about that length of time. The rule should be to first find the point of tolerance and then hold the dosage just below that point. The treatment should be begun just so soon as the diagnosis of syphilis can be made, whether the eruption has appeared or not. The initial lesion assumes one of three forms—an ulcerated sclerotic nodule, an eroded sclerotic nodule, or a dry nodule. The diagnosis can be made from the chancre alone in the last two cases, but when the lesion is ulcerative it is better to wait for secondaries or until the lesion changes into one of the other two forms. Enucleation of the lesion does not appear to prevent the development of secondaries. The advantage of giving mercury in primary syphilis is that it prevents or diminishes the eruption, with its attendant temporary or permanent disfigurement. For the local treatment of ulcerative lesions superficial caustic treatment is advocated. Ayres has found a saturated solution of potassium permanganate of most service. Also a 20 per cent. silver-nitrate solution is efficacious. A piece of cotton soaked in balsam of Peru is one of the best dressings. The use of tobacco may be allowed when any eruptions in the mouth have disappeared. It will not cause their reappearance if the patient is getting sufficient mercury. There is a consensus of opinion that the mercury given should circulate in the blood in minutely divided particles. When chemical combinations are given this is supposed to result from the components being broken up in the system. Ayres regards blue ointment as the most valuable of the preparations most frequently used for syphilis. It does not cause any intestinal trouble. The objection to it is that owing to its nastiness few patients will use it long. The back and loins are the best sites for the inunction, or failing that, those parts of the body where lymphatics are most freely distributed, viz., the inner sides of the arms, the axillary line of the chest, the inner sides of the thighs, and over the popliteal spaces, these areas to be used in rotation. Protiodide of mercury the author has found to be of very little use in the majority of cases, though sometimes it acts very well. It frequently upsets the digestive organs. Grey power is a mild preparation, and is sometimes borne where other preparations are not. Bichloride is very efficacious, but liable to cause digestive troubles. It may well be given in compound tincture of gentian. Ayres does not recommend iron to be given with mercury at first, as the two are then liable to produce an explosive salivation. Sometimes, however, symptoms will come under control with iron and mercury, but not with mercury alone. In very severe cases the hypodermic method of administration will control symptoms better than any other. A large portion of Ayres' paper is occupied with details of his experience with mercurol given in tabloid form. He has used it in 180 cases, and in private practice uses this drug to the exclusion of all others. Two or three grains given three times a day is the most that patients can stand or require. King Houchin [13] read a paper on the "Inunction Treatment of Syphilis," at the Balneological Society in October. He said that treatment as efficacious as that at Aix-la-Chapelle could be and was carried out at most of the British spas, and even in London. Great care had to be taken of the teeth. Chlorate of potash solution as a mouth-wash, together with a mild astringent, should be used frequently. A bath at 98° to 100° Fahr. was first taken and then the mercury rubbed in. He divided the body into five sections and rubbed the ointment into each of these on consecutive days, so that in five days the whole of the surface had been rubbed. This was repeated until 25 or 30 applications had been made. Two or three such courses at six-month intervals were usually sufficient to cure the patient. G. A. Leon, in the discussion, said that in tertiary cases several "courses" of daily inunction were necessary, each course extending over 50 to 150 days. A more astringent mouth-wash than chlorate of patash should be used, such as dilute acetate of lead and alum.

[12] Lancet, Oct. 19, 1901, p. 1037. [13] Lancet, Nov. 16, 1901, p. 1344

